# Cardiff Hospital and Viscount Tredegar

**Published:** 1913-03-22

**Authors:** 


### Cardiff Hospital and Viscount Tredegar.

SOME INTERESTING REMINISCENCES.

There is probably no institution for the welfare of the community in the counties of Glamorgan and Monmouth—and, indeed, in many places beyond their borders—that has not been assisted through the munificence of the late Viscount Tredegar, and of these the King Edward VII.'s Hospital, Cardiff, cherishes a long record of his personal consideration. The tribute paid by General Lee at the monthly meeting of the board of management of the hospital was followed by one from Colonel Bruce Vaughan, who truly said that it was not only the amount of Lord Tredegar's response to his numerous appeals that was valued, but also the infectious enthusiasm of his personality. As instancing the personal trouble which he took, it may be mentioned that some years ago an entertainment was promoted for the hospital which appeared likely to fail for want of social backing. The secretary wrote to Lord Tredegar explaining the circumstances and asking for his patronage. He received an answer by return of post that Lord Tredegar would attend the entertainment in person. Within a few days of this becoming known every single ticket was sold.

The following extracts from the records of his speeches at functions in connection with this hospital illustrate his fascinating method of public speaking. In proposing "Prosperity to the Cardiff Infirmary" at its first festival dinner, the Chairman said: "Many were present that night at great inconvenience, but he hoped not without some expense. The secretary would let them know about it presently;" and later, "Subscriptions given to the infirmary were less liable to imposition than any other form of charity. Nobody broke his leg or caught fever on purpose to get into hospital." In proposing a vote of thanks to Mr. Edmund Owen for his presence and address at the stone-laying of the new out-patient department, Lord Tredegar said that Mr. Owen had proved in his address that he could talk about other things than cutting off legs and taking out people's insides. He (Lord Tredegar) had expected to hear a scientific speech on the latest way in which they would take out somebody else's brain and put a better brain in its place. He believed this was quite possible. It was done every day, and he was sure a great number of people present at that meeting would be the better for it. A doctor in practice at a hospital saw more in one day than the ordinary practitioner who drove about the country in a closed carriage—which was sometimes irreverently called a "pill-box"—saw in a week. Again, at a distribution of "Lady Aberdare League" medals for shilling collectors for the hospital, he described his experiences at King Edward's Coronation. He was in a carriage all by himself in the middle of a long procession that did not always proceed at a smart pace. He had drawn aside his robes proudly to display the medals on his breast. These included the Queen Victoria Jubilee medal, the King's Coronation medal, and his Volunteer medal for long service. They made a great "show." As the procession passed along one old lady in the crowd shouted out, "Look there!"—indicating the speaker—"What a lot of lives he must have saved!" Referring to a diary, on this same occasion, of Morgan, of Coed-y-Goras, containing references to things that had occurred during 1724 to 1735 in the diarist's life, he could not find a word about a doctor or medicine. "No one," he added, "could keep a diary nowadays for any length of time without referring to a doctor's bill at some time or another."

Probably Lord Tredegar's last public appearance in connection with the Cardiff Infirmary (as King Edward VII.'s Hospital was at that time called) was in January 1911, when he again presided on the occasion of the second festival dinner. He then proposed the toast of the hospital in his usual vein. Alluding to the educational side of a hospital, he did not see how it was possible to have a really successful great hospital without an educational establishment attached to it. And later came a characteristic anecdote in reference to a recent controversy about doctors' fees. He once heard of a doctor who sent a bill to a leading tradesman in the town and received in reply a note to the effect that "You should treat me for nothing, because it was I who brought scarlet fever into the town." It is pathetic now to recall his Lordship's response to the toast of "The Chairman" on this occasion. The other day, he remarked, a doctor congratulated him on the state of his health and, after asking his age, said: "Well, you have no business to be alive." He forgot whether he considered it a compliment or not, but he thought, after the kind things that had just been said, he would try, with the assistance of the doctors, to live a little longer. The foregoing extracts emphasise unduly the late Viscount's humour. Those who were privileged to come within the sphere of his influence will also remember his wisdom, his chivalry, his tender humanity, and feel thankful for the inspiration of a good life.

